# ARID1A deficiency activates OSM-STAT3 axis in endometrial cancer, creating vulnerability to JAK/STAT3 inhibition

**DOI:** 10.7150/ijbs.129142

**Published:** 2026-02-11

**Authors:** Li-Jie Chen, Changxiang Shi, Eun Ju Yang, Guowen Ren, Shishi Tao, Yue Pu, Xiumei Zhang, Xin Shen, Changjie Wu, Joong Sup Shim

**Affiliations:** 1 Cancer Centre, Faculty of Health Sciences, University of Macau, Taipa, Macau SAR, China.; 2 Nanjing Key Laboratory of Female Fertility Preservation and Restoration, Nanjing Women and Children's Healthcare Institute, Women's Hospital of Nanjing Medical University (Nanjing Women and Children's Healthcare Hospital), Nanjing, 210004, China.; 3 Institute of Cancer Research, Shenzhen Bay Laboratory, Shenzhen, Guangdong, 518055, China.; 4 Guangdong Provincial Key Laboratory of Gastroenterology, Department of Gastroenterology, Nanfang Hospital, Southern Medical University, Guangzhou, 510515, China.; 5 MOE Frontiers Science Centre for Precision Oncology, University of Macau, Taipa, Macau SAR, China.

**Keywords:** endometrial cancer, synthetic lethality, ARID1A, OSM/STAT3/PLK1 axis

## Abstract

ARID1A, a key component of the SWI/SNF chromatin remodeling complex, is a tumor suppressor frequently inactivated in many cancer types, including endometrial cancer. Exploiting ARID1A deficiency has emerged as a therapeutic strategy in these types of cancer. We here employed a synthetic lethal drug screen for ARID1A and found that JAK/STAT3 pathway is a therapeutic vulnerability in ARID1A-deficient endometrial cancer. Inhibition of JAK/STAT3 selectively inhibited the growth of ARID1A deficient endometria cancer cells *in vitro* and in a mouse xenograft tumor model. Mechanistically, ARID1A deficiency activates JAK/STAT3 signaling through promoting the transcription of the pleiotropic cytokine Oncostatin M (OSM). Autocrine activation of JAK/STAT3 signal by OSM in ARID1A-deficient endometrial cancer cells promotes PLK1 levels, inducing mitotic abnormality. These cells are highly vulnerable to JAK/STAT3 and PLK1 inhibitors for mitotic arrest and death. ARID1A and OSM protein levels are inverse correlated in patients with endometrial cancer, where elevated OSM levels are associated with poor patient survival. Our study indicates that OSM-STAT3-PLK1 axis inhibition presents a new therapeutic approach for endometrial cancer with ARID1A loss.

## Introduction

Endometrial cancer (EC) is the sixth most common carcinoma in women 1, with its incidence rate increasing over the past decades 2. The standard surgical treatment for EC is a total hysterectomy with bilateral salpingo-oophorectomy. However, this surgical approach results in loss of fertility and is only suitable for early-stage EC 3. For advanced cases, pharmacological therapy becomes essential. The current first-line drug treatment includes chemotherapy agents such as carboplatin and paclitaxel, which are associated with severe side effects 4. Although mTOR inhibitors and immune checkpoint inhibitors have been developed for EC, they exhibit limited antitumor activity or significant treatment-related toxicity 5, 6. Immune checkpoint inhibitors like pembrolizumab and dostarlimab, approved by the US Food and Drug Administration (FDA) for mismatch repair-deficient advanced or relapsed endometrial cancer, show better efficacy but still result in grade 3-4 treatment-related toxicity in about 66% of patients 7. Therefore, developing new targeted therapies with high efficacy and low side effects for advanced EC is crucial.

AT-rich interactive domain 1A (ARID1A) is a tumor suppressor and is one of the most frequently mutated genes in endometrial cancer 8. ARID1A is a component of SWI/SNF chromatin remodeling complex, which alters nucleosome positioning and composition, thereby regulating the initiation and elongation of transcription for target genes 9. The human SWI/SNF complex exists in two forms: BRM-associated factors (BAF) and Polybromo-associated BAF (PBAF). Both forms contain one ATPase (SMARCA2/4), three core subunits (SMARCB1, SMARCC1, SMARCC2,) and an additional 8 to 11 accessory subunits. Within the BAF complex, ARID1A serves as the accessory subunit responsible for DNA binding 10. ARID1A is frequently mutated or epigenetically silenced in various cancers. Endometrial cancer ranks second in terms of ARID1A mutations, making ARID1A a crucial biomarker for this type of cancer.

In this study, we employed a synthetic lethality approach to pharmacologically target ARID1A loss in endometrial cancer and identified that JAK/STAT3 pathway as a therapeutic vulnerability in ARID1A-deficient endometrial cancer. The loss of ARID1A activates the transcription of the IL-6 family of cytokine Oncostatin M (OSM), which in turn stimulates the JAK/STAT3 pathway and its downstream signaling, including PLK1. Inhibition of JAK/STAT3 in ARID1A-deficient cells suppresses STAT3-PLK1 pathway, leading to abnormal spindle formation and mitotic catastrophe. Our findings suggest that targeting the OSM-STAT3 axis represents a new therapeutic strategy for treating endometrial cancer.

## Materials and Methods

### Cell lines and culture

HEC1B and AN3CA cell lines were cultured in MEM medium, KLE cells were cultured in DMEM/F12 medium, Ishikawa cells were cultured in DMEM medium, HCT116 cells were cultured in RPMI 1640 medium, and HEC1A cells were cultured in McCoy's 5A medium. All culture media were supplemented with 10% fetal bovine serum (FBS) and 1% penicillin/streptomycin (P/S). All the cell lines were obtained from American Type Culture Collection (ATCC) and cultured them in an incubator, set to 37 °C, added with 5% CO_2_. All culture media and supplements were acquired from Life Technologies (USA).

### Generation of ARID1A-isogenic cells

The CRISPR/Cas9 and HDR donor plasmid (Santa Cruz Biotechnology) were used to construct ARID1A^-/-^ cell. The CRISPR/Cas9 plasmid has a pool of three sgRNAs. Among them, sgRNA-1 target site and sgRNA-2 target site locate in exon2. SgRNA-3 target site locate in exon4. SgRNA sequence information was shown in Supplementary Table S2. The HDR donor plasmid contained red fluorescent protein gene and puromycin-resistant gene which were used to assess and select ARID1A^-/-^ single clones. The CRISPR/Cas9 and HDR donor plasmid were transfected to HEC1B cell by Lipofectamine 3000 (Thermo Fisher Scientific). Agarose gel electrophoresis, sanger sequence and western blot analysis were performed to prove the successful construction of HEC1B-ARID1A isogenic cell line. Sequencing primers targeting ARID1A exons 2 and 4 were designed to encompass all three sgRNA target sites, with their sequences listed in Supplementary Table S3.

### Epigenetics drug library and the kinase inhibitor library screening

Epigenetics Drug Library and the Kinase Inhibitor Library screening were purchased from Selleck Chemicals. The Epigenetics Drug Library contains 128 small molecule inhibitors of epigenetic proteins. The Kinase Inhibitor Library comprises 430 small-molecule inhibitors that selectively target a diverse array of druggable cellular targets. Both drug libraries were dispensed into 384-well plates using an 8-dose interplate dilution scheme with the concentration range from 13 nM to 30 μM (Epigenetics Drug Library) and from 9 nM to 20 μM (Kinase Inhibitor Library). HEC1B ARID1A^+/+^ and ARID1A^-/-^ cell were plated at a density of 1,000 cells/well directly into 384-well plates containing Epigenetics Drug Library and the Kinase Inhibitor Library. Following a 120-hour incubation at 37°C, cell viability was assessed using the AlamarBlue assay. The IC₅₀ of individual compounds was determined with GraphPad Prism 8 (GraphPad Software). Compounds exhibiting a selectivity index (SI=IC50^ARID1A+/+^/ IC50^ARID1A-/-^) greater than 2 were identified as potential synthetic lethality candidates.

### Cell viability assay

Cells were seeded in 96-well plates at 3,000 cells/well and allowed to adhere for 24 h prior to drug treatment. Cell viability was then assessed using an AlamarBlue assay. For this, a 10% (v/v) AlamarBlue solution—prepared by dissolving resazurin sodium salt (Sigma) to 0.025% (w/v) in sterile PBS (Gibco)-was added to the culture medium. After incubation at 37 ºC, fluorescence was measured (Ex560/Em590) using a SpectraMax M5 microplate reader (Molecular Devices, Sunnyvale, CA). Concurrently, cell images were acquired with an IncuCyte ZOOM system (Essen BioScience, Ann Arbor, MI).

### Tumor xenograft mouse model

All animal experiments were carried out base on ARRIVE guidelines, and approved by the Animal Research Ethics Committee of the University of Macau 11. For the xenograft mouse model, suspensions of the HEC1B-ARID1A isogenic cell pair in Matrigel (BD Biosciences, San Jose, CA) were used to inject into eight-week-old, female nude mice (3 × 10^6^ cell/mouse), in the left and right flanks. Three days after injection, we observed tumor volume is about 180 mm^3^, suggesting successful tumor xenografts. Then, the mice were randomly allocated into three groups (n=6 per group). They received daily intraperitoneal (I.P.) injections for 19 days, as follows: the control group was given the vehicle (0.9% NaCl, 5% DMSO, 5% Tween-80, and 5% PEG-400), while the treatment groups received stattic at either 5 mg/kg or 10 mg/kg, formulated in the same vehicle. Mouse body weight and tumor volume were monitored periodically throughout the study. Drug toxicity was assessed according to the changes of mice body weight. Tumor dimensions (long and short axes) were measured using a vernier caliper, and volumes were calculated with the modified ellipsoid formula: (long axis × short axis^2^ × π/6). At the experimental endpoint, mice were euthanized, tumors were harvested, weighed, and stored for subsequent immunoblot analysis.

### Cell cycle and apoptosis analysis

For cell cycle analysis, stattic-treated cells were harvested, washed with PBS, and fixed in pre-cooled ethanol (-20 ºC) overnight. After another PBS wash, cells were stained in the dark at 4 °C for 30 min using a solution containing PI (50 μg/ml), RNase A (100 μg/ml), and 0.1% Triton X-100 in PBS. For apoptosis analysis, cells were processed using the Alexa Fluor 488 Annexin V/Dead Cell Apoptosis Kit (Thermo Fisher Scientific) according to the manufacturer's instructions. Samples from both assays were analyzed on a BD Accuri C6 flow cytometer (BD Biosciences, CA), and data were processed with FlowJo software.

### Small interfering RNA (siRNA) silencing

All siRNAs were commercially synthesized (GENERAL BIOL), with sequences provided in Supplementary Table S4. For transfection, Lipofectamine RNAiMAX reagent and each siRNA were separately diluted in Opti-MEM medium, combined, and incubated for 5 min before being added to pre-seeded cells. Following transfection, knockdown efficiency was validated by immunoblotting, and phenotypic effects on cell viability were assessed via the AlamarBlue assay.

### Immunoblot analysis and antibodies

Whole-cell lysates were prepared using 2X Laemmli sample buffer, including 65.8 mM Tris-HCl (pH 6.8), 40% glycerol, 8% SDS, 0.04% bromophenol blue, while tumor tissue proteins were extracted with RIPA lysis buffer supplemented with protease inhibitors. Protein samples were separated by SDS-PAGE and transferred onto PVDF membranes. The membranes were then blocked and incubated overnight at 4 ºC with specific primary antibodies, followed by a 1-hour incubation with HRP-conjugated secondary antibodies at room temperature. Signal detection was performed using Clarity^TM^ Western ECL Substrate (Bio-Rad), and images were captured with a ChemiDoc^TM^ MP Imaging System (Bio-Rad). The information of primary antibodies was shown as follows: ARID1A (Santa Cruz, sc-32761, 250 kDa), STAT3 (Cell Signaling Technology, #9139s, 80 kDa), PSTAT3 (Cell Signaling Technology, #9145s, 80, 125 kDa), OSM (Abcam, ab133748, 22 kDa), leptin (Abcam, ab16227, 16 kDa), p-Histone H3(Ser10) (Santa Cruz, sc-8656-R, 17 kDa), PLK1 (Cell Signaling Technology, 4513s, 50 kDa).

### Reverse transcription and real-time quantitative PCR (RT-qPCR)

Total RNA was isolated using a fast extraction buffer (10 mM Tris pH 7.4, 0.25% Igepal CA-630, 150 mM NaCl) 12. Following extraction, mRNA was reverse-transcribed into cDNA using a High-Capacity cDNA Reverse Transcription Kit (Thermo Fisher Scientific). Quantitative PCR (qPCR) was then performed on a CFX96 Real-Time system (Bio-Rad) using iTaq Universal SYBR Green Supermix (Bio-Rad) and gene-specific primers (sequences in Supplementary Table S5).

### Immunofluorescence staining

Cells were seeded in 8-chamber slides (Lab-Tek II, Thermo Fisher Scientific) and fixed with 4% paraformaldehyde at 37 °C for 30 minutes. After permeabilization and blocking in 3% BSA with 0.1% Triton X-100 (RT, 30 min), cells were incubated with primary antibody at 4 °C overnight, followed by a 1-hour incubation at room temperature with Alexa Fluor 488- or 647-conjugated secondary antibodies. Following PBS washes, samples were mounted with DAPI-containing Fluoromount-G^TM^ (Thermo Fisher Scientific) and imaged using a Zeiss LSM 710 confocal microscope (Carl Zeiss, Thornwood, NY). The primary antibody includes STAT3 (Cell Signaling Technology, #9139s), PSTAT3 (Cell Signaling Technology, #9145s), p-Histone H3(Ser10) (Santa Cruz, sc-8656-R), α-tublin (Santa Cruz, sc-5286), PLK1 (Cell Signaling Technology, 4513s).

### Chromatin immunoprecipitation (ChIP) analysis

Chromatin immunoprecipitation (ChIP) was performed using the Imprint Chromatin Immunoprecipitation Kit (CHP1, Sigma-Aldrich) according to the manufacturer's protocol. Briefly, DNA and proteins were crosslinked with 1% formaldehyde, and chromatin was sheared to an appropriate size range using a Bioruptor sonicator (Diagenode, Denville, NJ). The sheared chromatin was then immunoprecipitated overnight at 4ºC with protein A-coated wells coupled with specific antibodies: anti-ARID1A (10 µg; Santa Cruz, sc-32761), anti-RNA polymerase II (1 µg; Sigma, from kit), or normal mouse IgG as a negative control. After reversing crosslinks and purifying the DNA, enriched DNA fragments were quantified by qPCR targeting the promoter regions of STAT3, OSM, and leptin. Primer sequences are listed in Supplementary Table S6.

### Immunohistochemistry (IHC)

The expression of ARID1A and OSM was evaluated by immunohistochemistry (IHC) on a human endometrial cancer tissue microarray (HUteA045PG01, Superchip, Shanghai, China). Briefly, following deparaffinization and antigen retrieval, sections were probed overnight at 4 °C with primary antibodies against ARID1A (1:100 dilution) or OSM (1:200). After washing, slides were incubated with an HRP-conjugated secondary antibody for 30 minutes at room temperature, and signals were developed using DAB substrate (Service, Wuhan, China). Stained images were captured with an NIS-element imaging system (Nikon, Japan).

### Statistical analysis

Statistical differences between control and experimental groups were assessed using Student's t-test or two-way ANOVA, as appropriate. Analyses were performed in GraphPad Prism 8, with a *P* value of less than 0.05 considered statistically significant.

## Results

### Combined drug library screens identified JAK/STAT3 inhibitors as synthetic lethal drugs for ARID1A-deficient endometrial cancer

**Figure 1:**
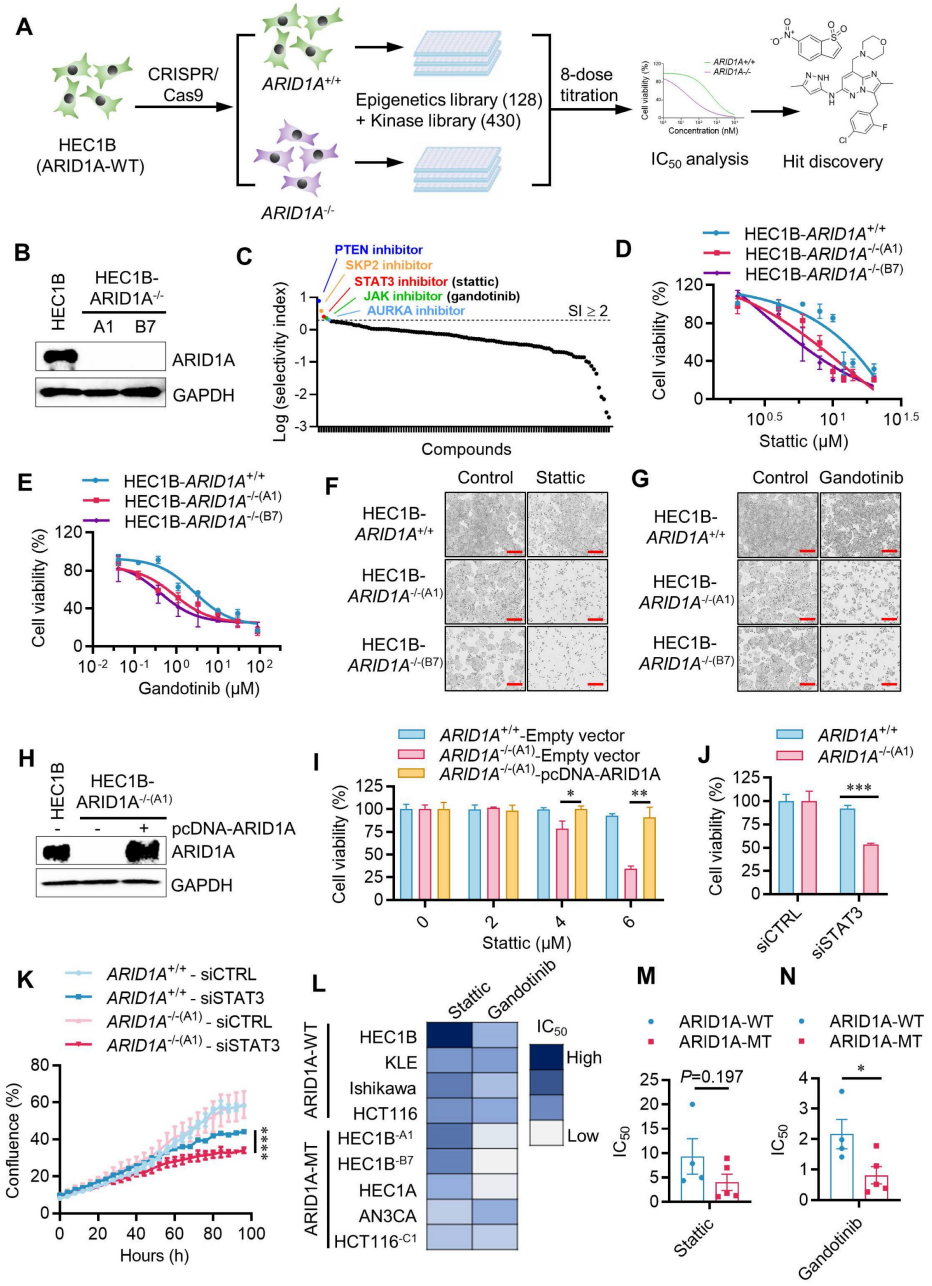
** Identification of synthetic lethality of ARID1A and JAK/STAT3 in endometrial cancer through drug library screening. A.** Schematic illustration of the synthetic lethality screenings with the Epigenetics Drug Library and the Kinase Inhibitor Library. **B.** Immunoblot analysis showing ARID1A knockout in A1 and B7 single clone.** C.** Drugs with selectivity index (SI)≥2 were selected as synthetic lethality candidates. SI=IC50^ARID1A+/+^/IC50^ARID1A-/-^.** D.** Synthetic lethality in HEC1B-ARID1A^-/-^ cell treated with stattic **(D, F)** and gandotinib **(E, G)** for 72h. The cell images were taken with IncuCyte ZOOM. Scale bar, 300 μm.** H.** Immunoblot analysis validated the ARID1A overexpression in HEC1B-ARID1A^-/-^ cell. **I.** Overexpressed ARID1A rescued the synthetic lethality mediated by stattic. Data are mean±sd. *P<0.05, **P<0.01. **J-K.** HEC1B ARID1A isogenic cell pair were transfected with STAT3-siRNA for 72 h. **J.** The quantitative analysis of synthetic lethality induced by STAT3-siRNA. Data are mean±sd. ***P<0.001. **K.** The growth curve of HEC1B ARID1A isogenic cell pair treated with STAT3-siRNA. ****P<0.0001. Two-way ANOVA test. L-**N.** IC50 test treated with stattic and gandotinib in endometrial cancer cell line. Data are mean±sd. *P<0.05.

To screen and identify ARID1A synthetic lethal targets, we generated an ARID1A-isogenic endometrial cancer cell pair using the CRISPR/Cas9 system. We introduced ARID1A gene knockout (KO) in HEC1B cells expressing wild-type ARID1A to mimic the functional loss of ARID1A observed in endometrial cancer. ARID1A-KO clones were verified through genomic PCR, sequencing, and Western blots of ARID1A (Fig. 1B, Supplementary Fig. S1A-C, Supplementary Fig. S2A-B). We used two homozygous ARID1A-KO clones (*ARID1A*^-/-^ clones A1 and B7) and their parental, ARID1A wild-type HEC1B cells for subsequent synthetic lethal target identification and validation. Given ARID1A's role in transcription regulation and cell signaling pathways, we selected two drug libraries for screening - Epigenetics Drug Library (128 drugs) and the Kinase Inhibitor Library (430 drugs)- which are functionally linked to ARID1A. We combined these libraries for the identification of ARID1A synthetic lethal targets. The screening was conducted in 384-well plates, using an 8-dose, inter-plate titration format to obtain IC_50_ values for each drug in each cell line (Fig. 1A). Drugs that selectively inhibited *ARID1A*^-/-^ cells over the *ARID1A*^+/+^ counterparts were identified as candidate synthetic lethal drugs. From the screen, we identified HOpic (PTEN inhibitor), SKPin C1 (SKP2 inhibitor), Stattic (STAT3 inhibitor), Gandotinib (JAK1/2/3 inhibitor) and MK-8745 (Aurora kinase-AURKA inhibitor) as top five synthetic lethal candidate drugs (Fig. 1C, Supplementary Table. S1). The synthetic lethal effects of all five candidates were validated in the ARID1A-isogenic cell pair. Among these drugs, we selected Stattic and Gandotinib for detailed exploration in this study as new synthetic lethal drugs, since their targets, JAK and STAT3, are both in the same pathway. Our validation study for HOpic and SKPin C1 indicated that the drugs exhibited synthetic lethal effects (data not shown). However, further investigation is needed to determine if their targets, PTEN and SKP2 respectively, are responsible for this synthetic lethality. Aurora kinase inhibitors have been reported to induce synthetic lethality with ARID1A loss in other types of cancer 13, supporting the feasibility of the synthetic lethal drug screening approach used in this study.

To explore the synthetic lethal interaction between ARID1A and JAK/STAT3 pathway, we first tested the effects of Stattic and Gandotinib in the parental *ARID1A*^+/+^ and two *ARID1A*^-/-^ HEC1B clones. Both Stattic and Gandotinib showed selective inhibition of *ARID1A*^-/-^ clones compared to the *ARID1A*^+/+^ counterparts (Fig. 1D-G). To determine if ARID1A deficiency contributes to the synthetic lethal effects of JAK/STAT3 inhibitors, we re-introduced wildtype ARID1A into *ARID1A*^-/-^ cells and tested its rescue effect on JAK/STAT3 inhibitor treatment.

The synthetic lethal effect of Stattic in *ARID1A*^-/-^ cells was significantly rescued when ARID1A was re-introduced (Fig. 1H-I). To verify whether the JAK/STAT3 pathway is responsible for the synthetic lethal effects of Stattic and Gandotinib, we depleted STAT3 expression in ARID1A-isogenic cells using siRNA and analyzed its synthetic lethal effect. Depletion of STAT3 expression in *ARID1A*^-/-^ cells indeed showed synthetic lethality, mirroring the drug effects (Fig. 1J-K, Supplementary Fig. S3). To test the generality of the synthetic lethal interaction between ARID1A and JAK/STAT3, we examined the inhibitor effects on various endometrial and colorectal cancer cells with different ARID1A mutation statuses. Our results show a clear trend of hypersensitivity of ARID1A-mutant cells to JAK/STAT3 inhibitors in endometrial cancer as well as other type of cancer cells (Fig. 1L-N). These data demonstrate that JAK/STAT3 inhibition is synthetic lethal with ARID1A deficiency in endometrial cancer cells.

### ARID1A deficiency promotes STAT3 signaling in endometrial cancer

**Figure 2:**
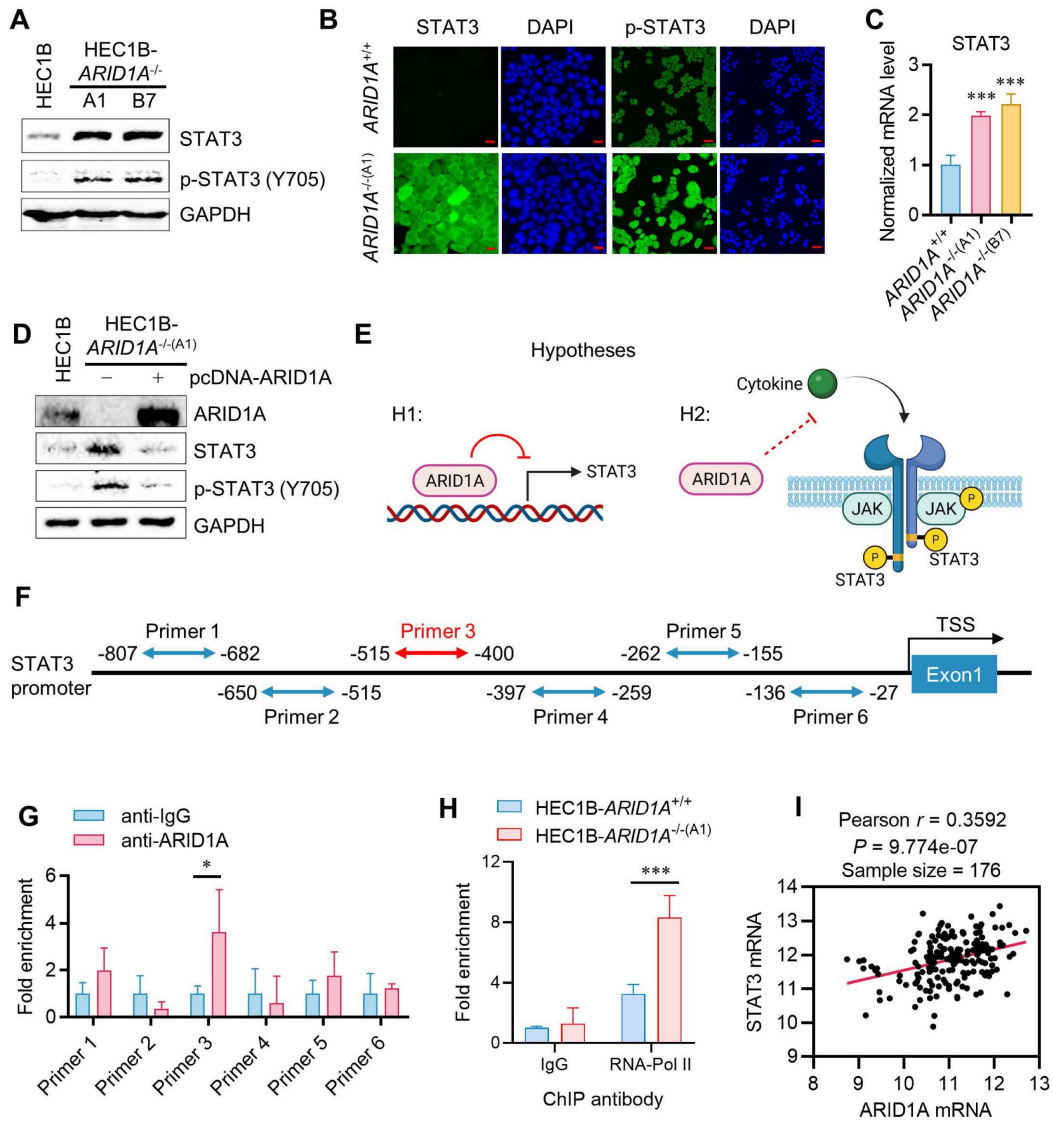
** Context-dependent transcription repression of STAT3 by ARID1A. A.** Immunoblot analysis of STAT3 signaling in HEC1B ARID1A isogenic cell pair. **B.** Immunofluorescence analysis of STAT3 and pSTAT3 in HEC1B ARID1A isogenic cell pair. Scale bar,20μm.** C.** The quantitative reverse-transcription PCR analysis of STAT3 in HEC1B ARID1A isogenic cell pair. Data are mean±sd. ***P<0.001. **D.** Immunoblot analysis of ARID1A overexpression in ARID1A^-/-^ cell. **E.** The two possibilities of STAT3 signaling deactivation in ARID1A^+/+^ cell. **F.** The designed primer in STAT3 promoter area for Chromatin immunoprecipitation (ChIP) experiment.** G.** The ChIP assay was performed using anti-ARID1A antibody. The chromatin was prepared from HEC1B-ARID1A^+/+^ cell. IgG as a control. Data are mean±sd. *P<0.05. **H.** The ChIP assay was performed using anti-RNA polymerase II (RNA poly II) antibody. The chromatin was prepared from ARID1A isogenic cell pair. IgG as a control. Data are mean±sd. ***P<0.001. **I.** Transcriptome profiling data of 176 endometrial cancer patients were obtained from The Cancer Genome Atlas (TCGA). The relative mRNA levels of STAT3 and ARID1A were analyzed. Pearson correlation analysis was used to assess statistical significance.

We next investigated the causal relationship between ARID1A deficiency and STAT3 signaling to explore the mechanism of synthetic lethality. Interestingly, *ARID1A^-/-^* cells expressed higher levels of total and phosphorylated STAT3 compared to *ARID1A^+/+^* cells (Fig. 2A-B). The STAT3 mRNA level was also increased in *ARID1A^-/-^* cells, suggesting that the elevated STAT3 level in *ARID1A^-/-^* cells is likely due to increased STAT3 transcription (Fig. 2C). Re-introducing ARID1A into *ARID1A^-/-^* cells reduced the levels of total and phosphorylated STAT3 to those similar to ARID1A wild-type cells (Fig. 2D). These data suggest that ARID1A negatively regulates STAT3 signaling through two possible mechanisms: (1) ARID1A represses STAT3 transcription, leading to an increase in total and phosphorylated STAT3 in ARID1A-deficient cells, and (2) ARID1A suppresses STAT3 phosphorylation through blockading upstream signaling, independently of its transcriptional regulation of STAT3 (Fig. 2E). To clarify these possibilities, we first tested whether ARID1A represses STAT3 transcription. Given that ARID1A is a component of the SWI/SNF complex and functions as a DNA-binding protein in transcription regulation, we investigated its direct binding to the STAT3 promoter and its role in the transcription regulation. We designed six primer pairs covering the STAT3 promoter region and performed chromatin immunoprecipitation (ChIP) using a ChIP-grade ARID1A antibody. The results indicated that ARID1A shows a significant binding to the primer 3 region (-515 to -400 from the transcription start site, TSS) of the STAT3 promoter (Fig. 2F-G). Furthermore, RNA Pol-II ChIP on the STAT3 promoter indicated that STAT3 is transcriptionally active in *ARID1A^-/-^* cells (Fig. 2H). These data suggest that ARID1A directly binds and represses the transcription of STAT3 in endometrial cancer cells. To verify whether the transcriptional regulation of STAT3 by ARID1A generally occurs in endometrial cancer cells, we analyzed the endometrial carcinoma patient cohort data (UCEC) from The Cancer Genome Atlas (TCGA). However, correlation analysis of the expression levels of ARID1A and STAT3 in the UCEC patient cohort showed that ARID1A and STAT3 expressions are positively correlated (Fig. 2I). This result indicates that the transcriptional regulation of STAT3 by ARID1A is likely a context-specific phenomenon, rather than a common occurrence across endometrial cancer patients. Additionally, the fact that both STAT3 and JAK inhibitors induce synthetic lethality in ARID1A-deficient endometrial cancer further suggests that ARID1A regulates the up-stream components of the JAK/STAT3 signaling pathway, rather than solely regulating STAT3 transcription.

### Oncostatin M (OSM) is a target of ARID1A and is responsible for JAK/STAT3 activation in endometrial cancer

**Figure 3:**
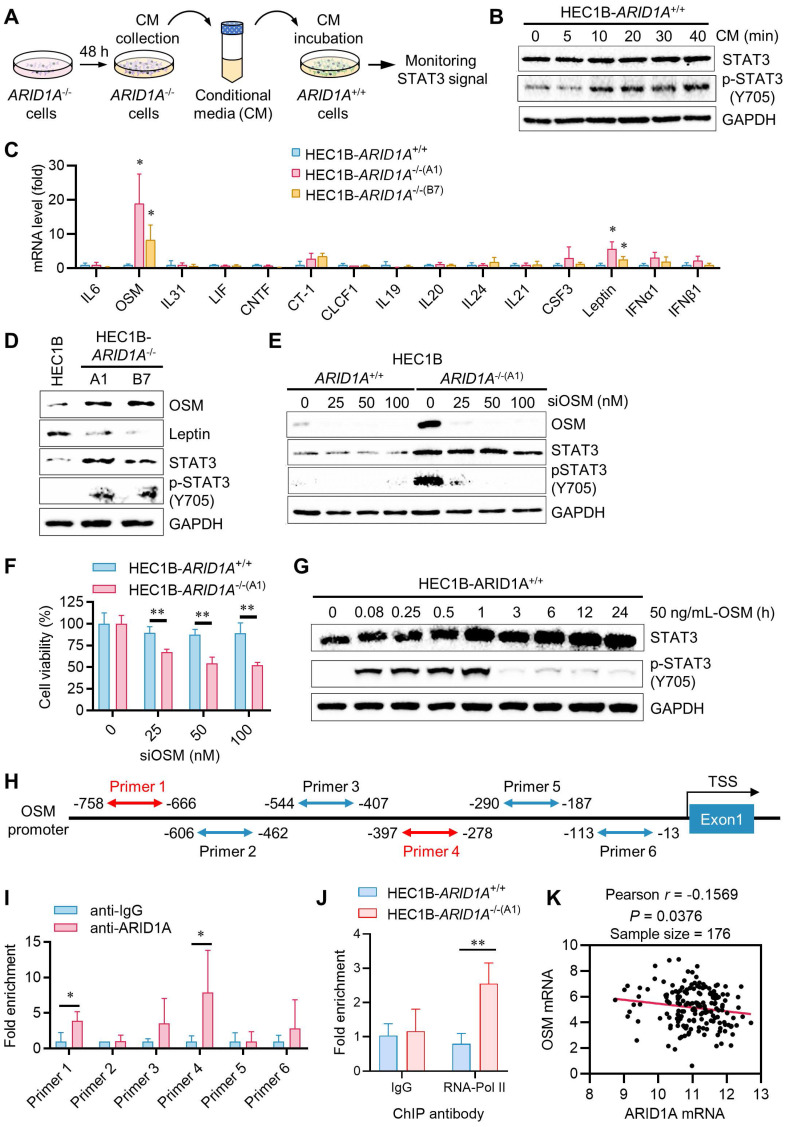
** Transcription repression of OSM and inhibition of the STAT3 signal by ARID1A. A.** The Schematic illustration of conditional medium (CM) preparation.** B.** The immunoblot analysis of the influence of CM on STAT3 signaling.** C.** The quantitative reverse-transcription PCR analysis of upstream cytokine of STAT3 signaling in HEC1B ARID1A isogenic cell pair. Data are mean±sd. *P<0.05 vs HEC1B-ARID1A^+/+^.** D.** The immunoblot analysis of the influence of upstream OSM and leptin on STAT3 signaling.** E.** The immunoblot analysis of the influence of silencing OSM on STAT3 signaling by transfecting OSM-siRNA to HEC1B ARID1A isogenic cell pair.** F.** Silencing OSM induced synthetic lethality in ARID1A^-/-^ cell. Data are mean±sd. **P<0.01.** G.** The immunoblot analysis of OSM active protein on STAT3 signaling.** H.** The designed primer in OSM promoter area for Chromatin immunoprecipitation (CHIP) experiment.** I.** The CHIP assay was performed using anti-ARID1A antibody. The chromatin was prepared from HEC1B-ARID1A^+/+^ cell. IgG as a control. Data are mean±sd. *P<0.05. **J.** The CHIP assay was performed using anti-RNA polymerase II (RNA poly II) antibody. The chromatin was prepared from ARID1A isogenic cell pair. IgG as a control. Data are mean±sd. **P<0.01. **K.** Transcriptome profiling data of 176 endometrial cancer patients were obtained from The Cancer Genome Atlas (TCGA). The relative mRNA levels of OSM and ARID1A were analyzed. Pearson correlation analysis was used to assess statistical significance.

JAK is activated by cytokine receptor dimerization in response to cytokine binding, such as interleukin-6 (IL-6) or interferon (IFN), which then transduces the signal to intracellular STAT3. Upon phosphorylation and dimerization, STAT3 dimers translocate to the nucleus and activate the transcription of target genes involved in various oncogenic cellular processes 14. Therefore, we sought to test upstream cytokines that can activate JAK/STAT3 in endometrial cancer and are also target genes of ARID1A. In fact, ARID1A is reported to regulate the transcription of IL-6 in ovarian cancer cells 15. To test this hypothesis, we first investigated whether components in the conditioned media (CM) from *ARID1A^-/-^* cell cultures could activate the JAK/STAT3 pathway. *ARID1A^+/+^* cells were incubated with the CM collected from *ARID1A^-/-^* cultures for various durations, and STAT3 phosphorylation was analyzed in *ARID1A^+/+^* cells (Fig. 3A). The CM from *ARID1A^-/-^* cells significantly activated STAT3 in *ARID1A^+/+^* cells after 10 minutes of incubation (Fig. 3B). These findings suggest that certain factors or cytokines secreted by *ARID1A^-/-^* cells are responsible for the autocrine activation of the JAK/STAT3 pathway. To identify the factors highly expressed in *ARID1A^-/-^* cells that activate JAK/STAT3, we analyzed the mRNA levels of all known upstream cytokines for JAK/STAT3, including the IL-6 family, IL-10 family, IL-21, G-CSF, Leptin, and IFNs. Among the cytokines examined, OSM (> 10-fold) and Leptin (3-5 folds) mRNAs were significantly upregulated in *ARID1A^-/-^* cells compared to *ARID1A^+/+^* cells (Fig. 3C). However, only OSM proteins, not Leptin proteins, were consistently increased in *ARID1A^-/-^* cells (Fig. 3D). We then explored the functional role of OSM in JAK/STAT3 signaling in endometrial cancer. Depletion of OSM using siRNA significantly suppressed STAT3 phosphorylation in *ARID1A^-/-^* cells (Fig. 3E), leading to reduced cell growth (Fig. 3F).

Additionally, adding recombinant OSM protein to *ARID1A^+/+^* cell culture media strongly activated the STAT3 pathway in *ARID1A^+/+^* cells (Fig. 3G). In contrast, adding Leptin did not activate the STAT3 pathway in *ARID1A^+/+^* cells (Supplementary Fig. S4A). These results suggest that OSM expression is negatively regulated by ARID1A and its upregulation is responsible for JAK/STAT3 activation in *ARID1A^-/-^* cells. We then investigated whether ARID1A, as a key component of the nucleosome remodeler, directly regulates OSM transcription in endometrial cancer cells. We designed six primer pairs covering the OSM gene promoter and performed ChIP experiments using an ARID1A antibody (Fig. 3H). Our results showed that ARID1A exhibited the most significant binding to the primer 4 (-397 to -278 from TSS) region on the OSM promoter (Fig. 3I). RNA Pol-II ChIP experiments indicated that OSM is transcriptionally more active in *ARID1A^-/-^* cells (Fig. 3J). However, no significant binding of ARID1A was observed on the Leptin gene promoter (Supplementary Fig. S4B-C), suggesting that the upregulation of Leptin mRNA in *ARID1A^-/-^* cells is likely an indirect mechanism of expression regulation. Lastly, analysis of a UCEC patient cohort revealed a weak negative correlation between ARID1A and OSM expression (Fig. 3K), suggesting that this regulatory relationship may also operate broadly in endometrial cancer. Together, these data indicate that OSM is a target of ARID1A for transcriptional repression and is a key cytokine responsible for JAK/STAT3 activation in ARID1A-deficient endometrial cancer.

### STAT3 inhibition induces mitotic arrest in* ARID1A^-/-^* cells by suppressing PLK1

**Figure 4:**
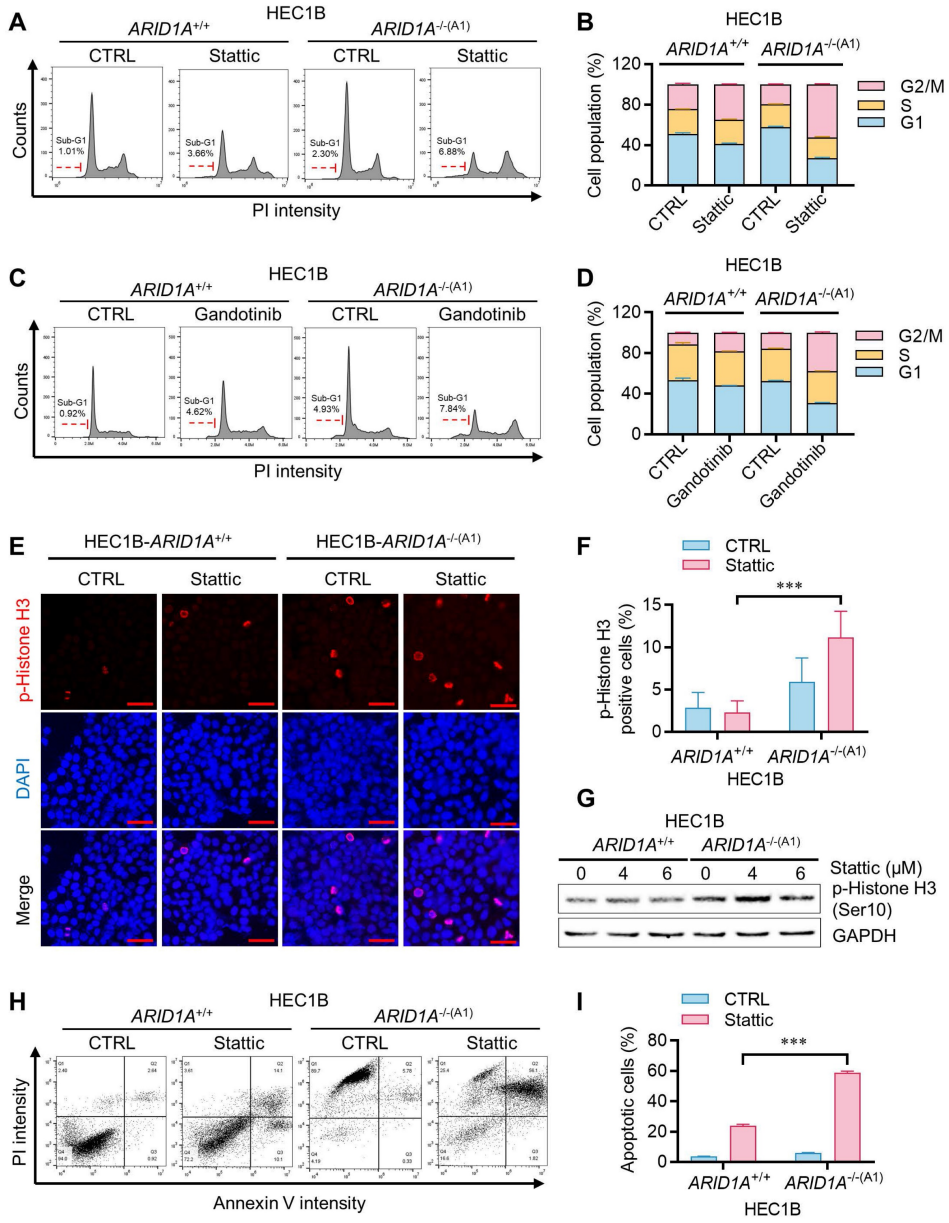
** Induction of mitotic arrest and apoptosis in ARID1A^-/-^ cells by STAT3 inhibition. A-D.** Cell cycle analyses of ARID1A isogenic HEC1B cells treated with stattic (**A**) and gandotinib (**C**). **B.** Percentage of cell populations in each cell cycle phase treated with stattic (**B**) and gandotinib (**D**). Error bars represent sd. **E.** Induction of mitosis in ARID1A^-/-^ cell by stattic. Immunofluorescence analysis of phosphorylation Histone H3 (Red) and DAPI (blue) after treatment with stattic 24h. Scale bars,50μm. **F.** Percentage of p-Histone H3 positive cells (%) in HEC1B ARID1A isogenic cell pair after treatment with stattic 24h. Data are mean±sd. ***P<0.001. **G.** The immunoblot analysis of p-Histone H3 in HEC1B ARID1A isogenic cell pair after treatment with stattic 24h. **H.** Apoptosis analyses of ARID1A isogenic HEC1B cells treated with stattic. Note that control *ARID1A*^-/-^ cells appeared PI-positive/Annexin V-negative due to RFP signals from the HDR donor plasmid used for ARID1A-knock out clone selection, which interfered with PI detection. Apoptosis quantification was therefore restricted to Annexin V-positive cells. **I.** The percentage of apoptotic cells were quantitated. Data are mean±sd. ***P<0.001.

We next investigated how JAK/STAT3 inhibition leads to synthetic lethality in ARID1A-deficient endometrial cancer cells. Cell cycle analysis of the ARID1A-isogenic HEC1B pair revealed that Stattic and Gandotinib preferentially induced G2/M cell cycle blockade in *ARID1A^-/-^* cells (Fig. 4A-D). The sub-G1 population also increased preferentially in *ARID1A^-/-^* cells treated with these drugs. Mitotic index analysis, detecting phosphorylated histone H3, indicated that the STAT3 inhibitor induced mitotic arrest preferentially in *ARID1A^-/-^* cells (Fig. 4E-G). These effects of JAK/STAT3 inhibitors led to selective apoptotic cell death in ARID1A-deficient endometrial cancer cells (Fig. 4H-I).

**Figure 5:**
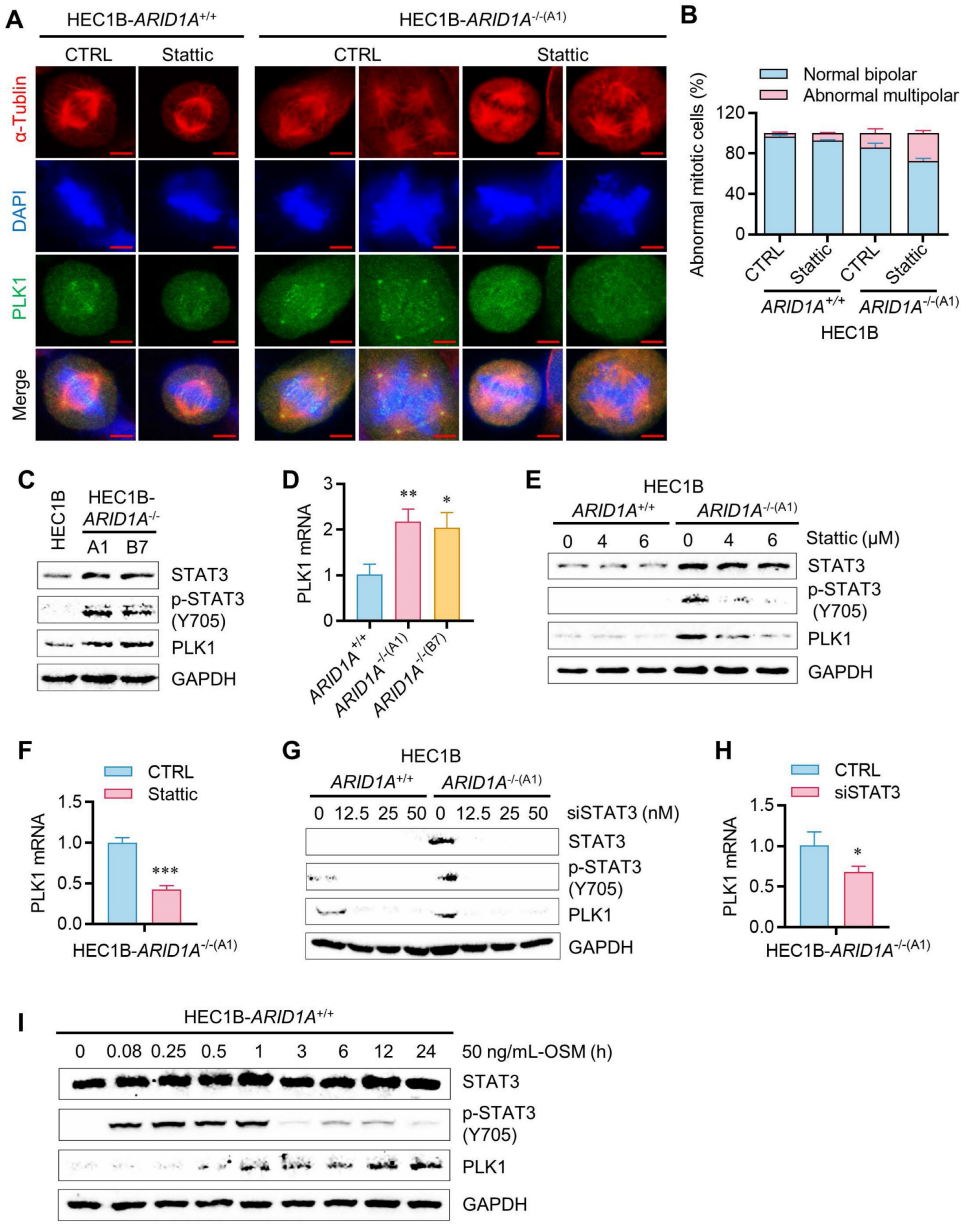
** Regulation of STAT3-PLK1 axis by ARID1A in endometrial cancer cells. A.** Induction of multipolar spindle in ARID1A^-/-^ cell by stattic. Immunofluorescence analysis of α-tublin (red), DAPI (blue) and PLK1 (green) after treatment with stattic. Scale bars,5μm.** B.** Percentage of abnormal mitotic cells (%) in HEC1B ARID1A isogenic cell pair after treatment with stattic. Data are mean±sd.** C.** The immunoblot showing STAT3 signaling activation and PLK1 upregulation in ARID1A^-/-^ cell.** D.** The quantitative reverse-transcription PCR analysis of PLK1 in HEC1B ARID1A isogenic cell pair. Data are mean±sd. *P<0.05, **P<0.01 vs HEC1B-ARID1A^+/+^. **E-H.** The inhibition of PLK1 protein **(E, G)** and mRNA **(F, H)** level by stattic and STAT3-siRNA. Data are mean±sd. *P<0.05, ***P<0.001.** I.** The immunoblot analysis of OSM active protein on STAT3-PLK1 signaling.

Given the high activation of STAT3 signaling in ARID1A-deficient endometrial cancer cells, we hypothesized that activated STAT3 might render these cells dependent on STAT3 function or oncogene addiction in mitotic processes. STAT3 has been linked to mitotic processes through its transcriptional regulation of PLK1 16, a key mitotic serine/threonine kinase involved in centrosome maturation, spindle formation, APC activation, and cytokinesis. More recently, STAT3 was reported to physically interact with stathmin and PLK1, regulating spindle-microtubule stability and centrosome clustering during mitosis 17. To investigate the role of JAK/STAT3 activation and the effect of its inhibition on mitotic processes in ARID1A-deficient cells, we analyzed mitotic phenotypes in the ARID1A-isogenic HEC1B pair treated with or without STAT3 inhibitor. ARID1A-deficient cells exhibited increased mitotic abnormalities, such as multipolar spindles, compared to ARID1A wildtype cells (Fig. 5A-B). STAT3 inhibition significantly exacerbated these mitotic abnormalities in *ARID1A^-/-^* cells. PLK1 protein and mRNA expressions were elevated in *ARID1A^-/-^* cells along with STAT3 phosphorylation (Fig. 5C-D). Stattic treatment or siRNA depletion of STAT3 expression reduced PLK1 levels, particularly in *ARID1A^-/-^* cells (Fig. 5E-H). The up-regulation of PLK1 expression in *ARID1A^-/-^* cells and its down-regulation by STAT3 inhibition occurred at the transcriptional level (Fig. 5D-H), suggesting that activated STAT3 in ARID1A-deficient cells stimulates PLK1 transcription. We further investigated whether elevated PLK1 in *ARID1A^-/-^* cells was due to OSM-mediated STAT3 activation. Incubation of *ARID1A^+/+^* cells with OSM time-dependently elevated PLK1 levels along with STAT3 phosphorylation (Fig. 5I). These data indicate that ARID1A loss de-represses OSM transcription, inducing cytokine receptor-mediated STAT3 activation, followed by PLK1 transcriptional activation in ARID1A-deficient endometrial cancer cells. In this context, STAT3 inhibition strongly suppresses PLK1 expression. Since centrosome separation and chromosome segregation are highly sensitive processes requiring sophisticated regulation, dysregulation of central mitotic kinases like PLK1 can be detrimental to faithful bipolar spindle formation and proper mitosis 18. Abnormal PLK1 expression or activity can lead to mitotic errors, resulting in chromosomal instability and potentially contributing to tumorigenesis or mitotic catastrophe. Therefore, dysregulation of PLK1 expression in *ARID1A^-/-^* cells and its inhibition by STAT3 inhibitors likely caused spindle and centrosome abnormalities in these cells.

### Synthetic lethality of ARID1A and STAT3 is mediated by PLK1

**Figure 6:**
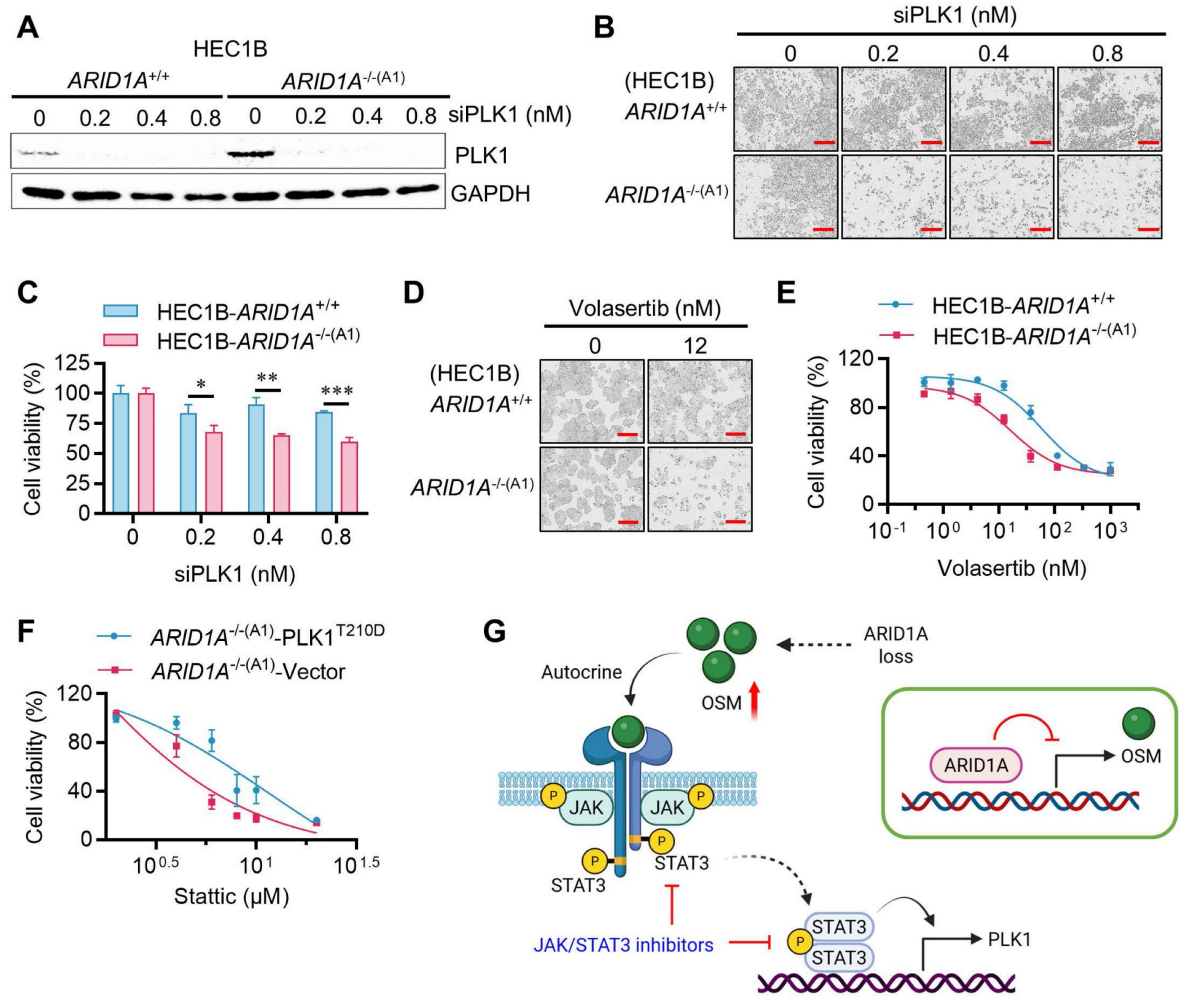
** PLK1 mediates the synthetic lethality of ARID1A and STAT3. A.** The immunoblot analysis of PLK1 after transfected PLK1-siRNA to HEC1B ARID1A isogenic cell pair.** B-E.** Synthetic lethality induction in HEC1B-ARID1A^-/-^ cell treated with PLK1-siRNA **(B, C)** and PLK1 inhibitor **(D, E)**. The cell images were taken with IncuCyte ZOOM. Scale bar, 300 μm. Data are mean±sd. *P<0.05, **P<0.01, ***P<0.001.** F.** PLK1 overexpression reversed the synthetic lethality in ARID1A^-/-^ cell treated with stattic.** G.** A working model showing that ARID1A deficiency activates OSM-STAT3-PLK1 axis in endometrial cancer. Inhibiting JAK/STAT3 signal induces synthetic lethality in ARID1A-deficient endometrial cancer (Created in BioRender. Shim, J. (2025) https://BioRender.com/xetqnmq)

If PLK1 is a critical mediator of STAT3 inhibitor-induced G2/M arrest and mitotic abnormalities in ARID1A-deficient cells, then inhibiting PLK1 may also induce synthetic lethality in ARID1A-deficient endometrial cancer cells. We therefore investigated the role of PLK1 in the synthetic lethality of ARID1A and STAT3. siRNA depletion of PLK1 in the ARID1A-isogenic cell pair selectively inhibited the growth of *ARID1A^-/-^* cells (Fig. 6A-C). Volasertib, a small molecule inhibitor of PLK1, also exhibited synthetic lethal effects in *ARID1A^-/-^* cells (Fig. 6D-E). Next, we overexpressed a constitutively active form of PLK1 (PLK1-T210D) in ARID1A-deficient cells and analyzed its rescue effect on the synthetic lethality induced by STAT3 inhibition. Overexpression of the active form PLK1-T210D in *ARID1A^-/-^* cells successfully rescued the synthetic lethal effect of Stattic (Fig. 6F, Supplementary Fig. S5A-B), indicating that PLK1 plays a key role in the synthetic lethality between ARID1A and STAT3. These data suggest that ARID1A deficiency promotes OSM transcription, leading to autocrine stimulation of JAK/STAT3 signaling. Hyperactivation of JAK/STAT3 in ARID1A deficient cells promotes transcriptional activation of PLK1, resulting in mitotic abnormalities. This phenomenon renders cells vulnerable to STAT3 or PLK1 inhibition, inducing mitotic arrest and cell death (summarized in Fig. 6G).

### STAT3 inhibition is synthetic lethal with ARID1A deficiency *in vivo*

**Figure 7:**
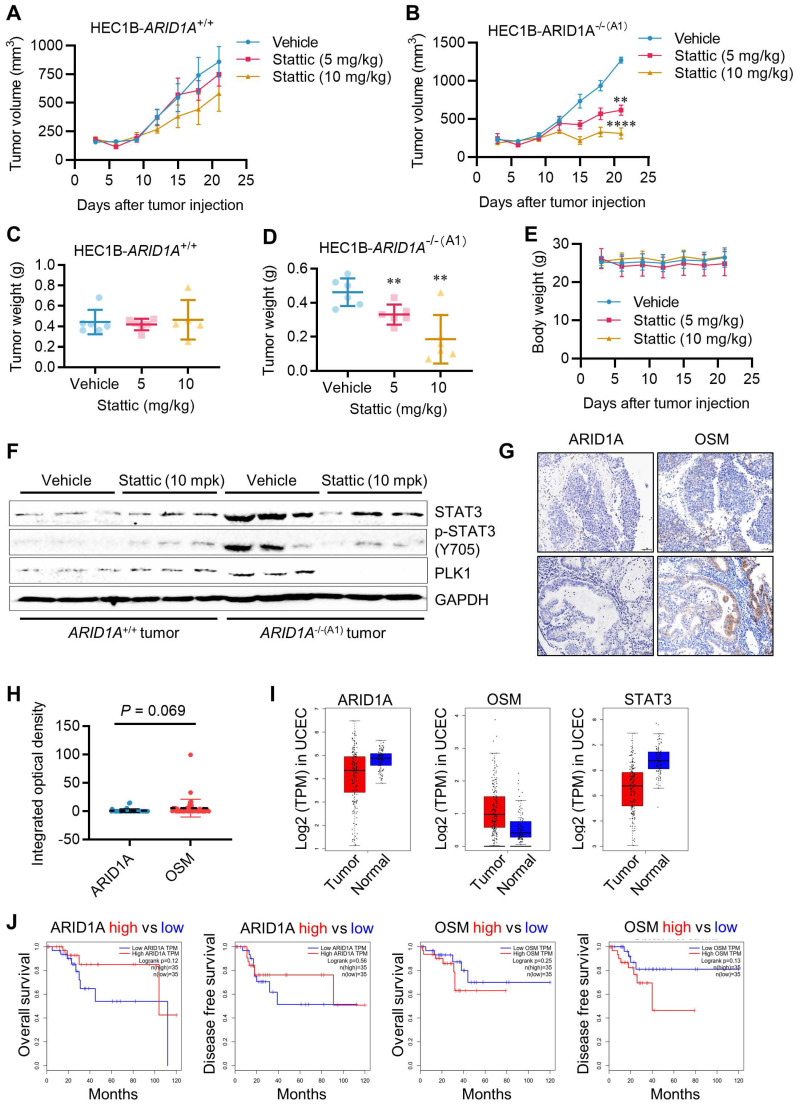
** Synthetic lethality of ARID1A and STAT3 *in vivo*. A-B.** Tumor growth curve after intraperitoneal injection of control or stattic in nude mice bearing ARID1A^+/+^**(A)** and ARID1A^-/-^**(B)** xenografts. Data are mean±sd. **P<0.01. ****P<0.0001. Two-way ANOVA test. **C-D.** Tumor weight in nude mice bearing ARID1A^+/+^**(C)** and ARID1A^-/-^**(D)** xenografts at 21 days after tumor injection. Data are mean±sd. **P<0.01. **E.** Body weight in nude mice in the period of treatment with stattic.** F.** Immunoblot analysis of STAT3, pSTAT3 and PLK1 expression in HEC1B ARID1A isogenic tumor pair. **G.** Representative ARID1A and OSM immunohistochemical (IHC) staining in endometrial tumors. Scale bars, 50 μm. **H.** The Integrated optical density (IOD) data of 45 endometrial tumor tissues were quantified with ImageJ. The relative expression levels of ARID1A and OSM were analyzed. Data are mean±sd. **I.** Transcriptome profiling data of 174 endometrial cancer patients and 91 normal patients were obtained from The Cancer Genome Atlas (TCGA). The mRNA levels of ARID1A, OSM and STAT3 were analyzed. **J.** Transcriptome profiling data of endometrial cancer patients were obtained from TCGA. The Survival analysis of ARID1A and OSM were analyzed.

To validate the synthetic lethal effects of STAT3 inhibition on ARID1A-deficient endometrial cancer *in vivo*, we conducted tumor xenograft experiments in mice bearing ARID1A-isogenic tumor pairs. *ARID1A^+/+^* and *ARID1A^-/-^* HEC1B cells were implanted bilaterally in nude mice, and Stattic (5 and 10 mg/kg) was administered intraperitoneally. As a result, Stattic had a marginal effect on the growth of *ARID1A^+/+^* tumors (Fig. 7A, C), while it significantly suppressed the growth of *ARID1A^-/-^* tumors at both doses (Fig. 7B, D). No significant toxicity was observed at the tested doses of Stattic (Fig. 7E). Analysis of tumor tissues revealed that phosphorylated STAT3 and PLK1 levels were highly elevated in *ARID1A^-/-^* tumors and were significantly down-regulated upon treatment with the STAT3 inhibitor (Fig. 7F). These results are consistent with our *in vitro* observations. We also analyzed the clinical significance of ARID1A and OSM levels in tumor samples from endometrial cancer patients. The results showed a trend of higher expression of OSM than that of ARID1A in the patients (Fig. 7G-H). This observation aligns with the UCEC cohort data from TCGA showing that ARID1A level is lower in tumor than in normal tissues, while OSM level was higher in tumor than in normal tissues (Fig. 7I). These findings suggest that ARID1A generally exerts a negative regulatory effect on OSM expression in endometrial cancer patients. Interestingly, STAT3 levels are lower in tumor tissues compared to normal tissues, and this reduction is positively correlated with ARID1A expression patterns. This observation supports the notion that ARID1A-mediated regulation of STAT3 transcription, as previously identified, is likely context-dependent. To further explore the clinical relevance, we analyzed the association between ARID1A and OSM expression levels and patient survival outcomes. Although the results did not reach statistical significance—primarily due to the limited sample size—a clear trend emerged: higher ARID1A expression is associated with improved patient survival, whereas elevated OSM levels correlate with poorer survival outcomes (Fig. 7J).

## Discussion

Endometrial cancer treatment faces several challenges that need to be addressed to improve patient outcomes. Specifically, surgery can lead to menopause and infertility, while radiation and chemotherapy often result in toxic side effects, significantly affecting the patient's quality of life 5, 19, 20. Personalized medicine tailored to individual genetic profiles is still a developing area and not widely available 21-23. Consequently, there is a pressing need to develop more targeted therapies based on tumor biomarkers. This study employed a synthetic lethality approach to identify specific druggable targets for endometrial cancer characterized by the loss of ARID1A, one of the most frequently mutated tumor suppressors in this cancer type. From the screening of combined drug libraries, we identified JAK/STAT3 inhibitors as synthetic lethal drugs in ARID1A-deficient endometrial cancer. Our mechanistic study revealed that ARID1A, a component of the SWI/SNF nucleosome remodeling complex, controls the level of the pleiotropic cytokine OSM by repressing its transcription in endometrial cancer. ARID1A deficiency in endometrial cancer activates OSM transcription, leading to autocrine stimulation of receptor-mediated JAK/STAT3 activation. This further activates the STAT3 target gene, PLK1, causing abnormal mitotic phenotypes. These events create cellular vulnerability to inhibitors of JAK/STAT3 and PLK1 in endometrial cancer cells. Lastly, mouse xenograft models and patient clinical association analyses verified the OSM-STAT3 axis as a therapeutic vulnerability in ARID1A-deficient endometrial cancer.

ARID1A is recognized as an emerging tumor suppressor, with its mutational deficiencies being linked to various cancers 8, 24. Notably, gynecological cancers exhibit high mutation rates in ARID1A, with frequencies ranging from 40-67% in ovarian cancer and 42% in endometrial cancer 25-27. ARID1A deficiency can result in various impacts on cellular processes, including dysregulation of gene transcriptions 28, defects in DNA damage responses 29, and abnormalities in the tumor microenvironment and signaling pathways 30, 31. As a crucial component of the SWI/SNF complex, ARID1A, with its DNA binding capability, typically targets SWI/SNF complexes to enhancers, where they cooperate with transcription factors to regulate gene transcriptions. In cells deficient in ARID1A, the function of SWI/SNF is maintained by ARID1B, but the targeting and enhancer control functions of SWI/SNF are impaired, resulting in widespread dysregulation of gene transcription 32. ARID1A also plays a role in regulating histone acetylation and deacetylation by recruiting histone acetyltransferase (HAT), bromodomain and extra-terminal motif (BET) proteins, or histone deacetylase (HDAC) to the enhancer or promoter regions of target genes. By influencing chromatin compaction or maintaining an open chromatin conformation, ARID1A epigenetically regulates the transcription of its target genes 33, 34. As a result, cells lacking ARID1A show dysregulation of several key oncogenes and tumor suppressors, which contributes to tumor initiation and progression. Identifying driver genes whose expressions are controlled by ARID1A and the SWI/SNF complex offers a unique opportunity to develop druggable targets in cancers with ARID1A deficiency. For example, ARID1A positively regulates the transcription of PIK3IP1, a negative regulator of PI3K/AKT signaling, which is repressed by the EZH2 methyltransferase. In ARID1A-deficient cells, PIK3IP1 levels are significantly reduced due to H3K27Me3 hypermethylation on its promoter by EZH2. Consequently, EZH2 inhibition has shown strong synthetic lethality in ARID1A-deficient ovarian cancer cells 35. Additionally, our group previously identified Aurora kinase A (AURKA) as a target of ARID1A for transcriptional repression in colorectal cancer cells. Colorectal cancer cells lacking ARID1A exhibit elevated AURKA levels, leading to hyperactivation of CDC25C and CDK2, which promotes G2/M transition and cellular oncogene addiction. Therefore, inhibition of the AURKA-CDC25C axis induces synthetic lethality in ARID1A-deficient colorectal cancer cells 13. In this study, we found that inhibiting the JAK/STAT3 pathway is synthetic lethal with ARID1A deficiency in endometrial cancer. Mechanistic studies revealed that OSM, a cytokine upstream of the JAK/STAT3 signal, is transcriptionally repressed by ARID1A, and its upregulation is linked to poor patient survival in endometrial cancer. ARID1A deficiency activates JAK/STAT3 and PLK1 signaling by promoting OSM transcription, creating a cellular dependency on this oncogenic pathway. We also observed that ARID1A binds to the STAT3 promoter and represses its transcription in endometrial cancer cells. However, this phenomenon appears to be context-specific, as analysis of a large cohort of endometrial cancer patients showed a negative correlation between ARID1A and OSM expressions, but not between ARID1A and STAT3. Recently, Amara et al. reported that JAK/STAT3 signaling is therapeutic vulnerability in SMARCB1-deficient bladder cancer 36. Their study demonstrated that SMARCB1 binds to the STAT3 promoter region and represses its transcription. SMARCB1-deficient cells exhibit an activated IL-6/JAK/STAT3 axis, making them vulnerable to JAK/STAT3 signaling inhibitors. This finding aligns with our results and suggests tissue-specific regulation of STAT3 transcription by the SWI/SNF complex, such as in bladder cancer. Our study indicates that JAK/STAT3 activation in ARID1A-deficient endometrial cancer is mediated by the transcriptional regulation of OSM.

The Janus kinase (JAK)/signal transducer and activator of transcription 3 (STAT3) signaling pathway is crucial for various biological processes, including immune function, cell growth, differentiation, and apoptosis 37. The pathway is activated when a ligand, such as a cytokine, binds to its corresponding receptor on the cell surface. This binding triggers receptor dimerization, followed by the sequential phosphorylation and activation of JAK and STAT3. Once phosphorylated and dimerized, STAT3 dimers translocate to the nucleus, where they activate the transcription of target genes involved in various oncogenic cellular processes, including cell cycle regulation and apoptosis 38, 39, angiogenesis 40, invasion and migration 41-43, immunosurveillance 44, and the maintenance of cancer stem cells 45. Consequently, several JAK/STAT3 inhibitors have been approved or are in clinical development for treating autoimmune diseases and certain cancers, such as myeloid leukemia. Notably, due to their role in cytokine signaling, JAK inhibitors are primarily approved for treating rheumatoid arthritis. Our study indicates that targeting JAK/STAT3 in ARID1A-deficient endometrial cancer offers a novel personalized treatment strategy for these patients. Therefore, it is essential to explore drug repurposing of approved JAK inhibitors for treating endometrial cancer with ARID1A deficiency as a personalized therapeutic approach.

## Supplementary Material

Supplementary figures and tables.
