# Supplementary material for: ARID1A deficiency activates OSM-STAT3 axis in endometrial cancer, creating vulnerability to JAK/STAT3 inhibition
Source: Int J Biol Sci. 2026 Feb 11;22(5):2638–53. doi: 10.7150/ijbs.129142 (PMC12965241; doi:10.7150/ijbs.129142)
Supplement: Supplementary file 1 — Supplementary figures and tables. [file ijbsv22p2638s1.pdf]

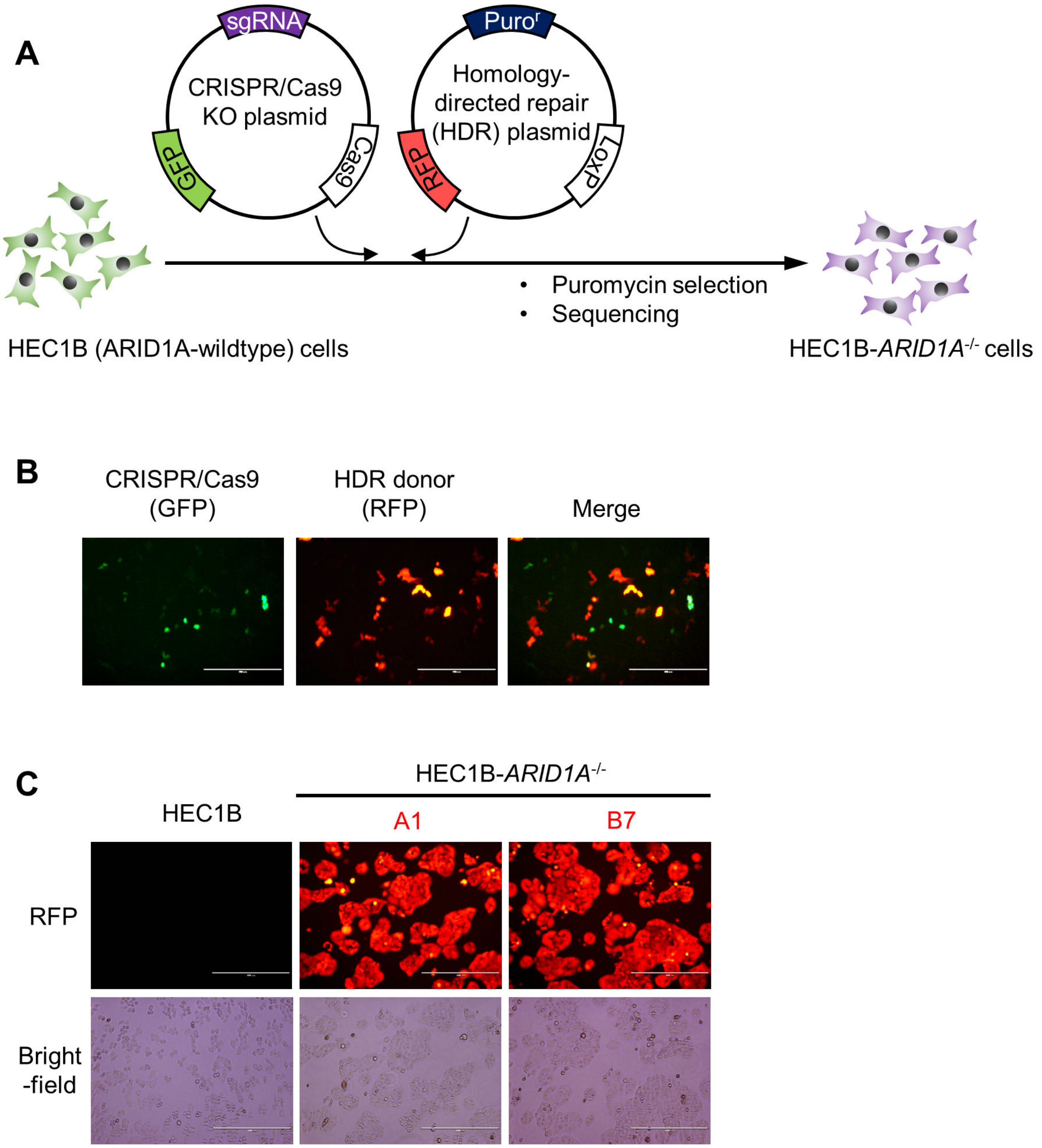

Supplementary Figure S1. Generation of ARID1A knockout (KO) HEC1B cells. A. HEC1B cells were transfected with CRISPR/Cas9 plasmids containing green fluorescence protein (GFP), 3 sgRNAs targeting ARID1A gene and a homology-directed repair (HDR) donor plasmid containing red fluorescence protein (RFP) and puromycin resistant gene (Puro^r). After 72 h of transfection, puromycin was added. After more than 80% of cell carry RFP, the cells were trypsinized and plated in 96 well plate at 0.5-1 cell each well for clone selection. Single clone was selected after they form a colony. **B.** Transfection efficiency was assessed with GFP (CRISPR/Cas9) and RFP (HDR donor plasmid). Scale bar, 400 μm. **C.** After 2 weeks of selection and clone isolation, *ARID1A*$^{-/-}$ clones have RFP only. Scale bar, 400 μm.

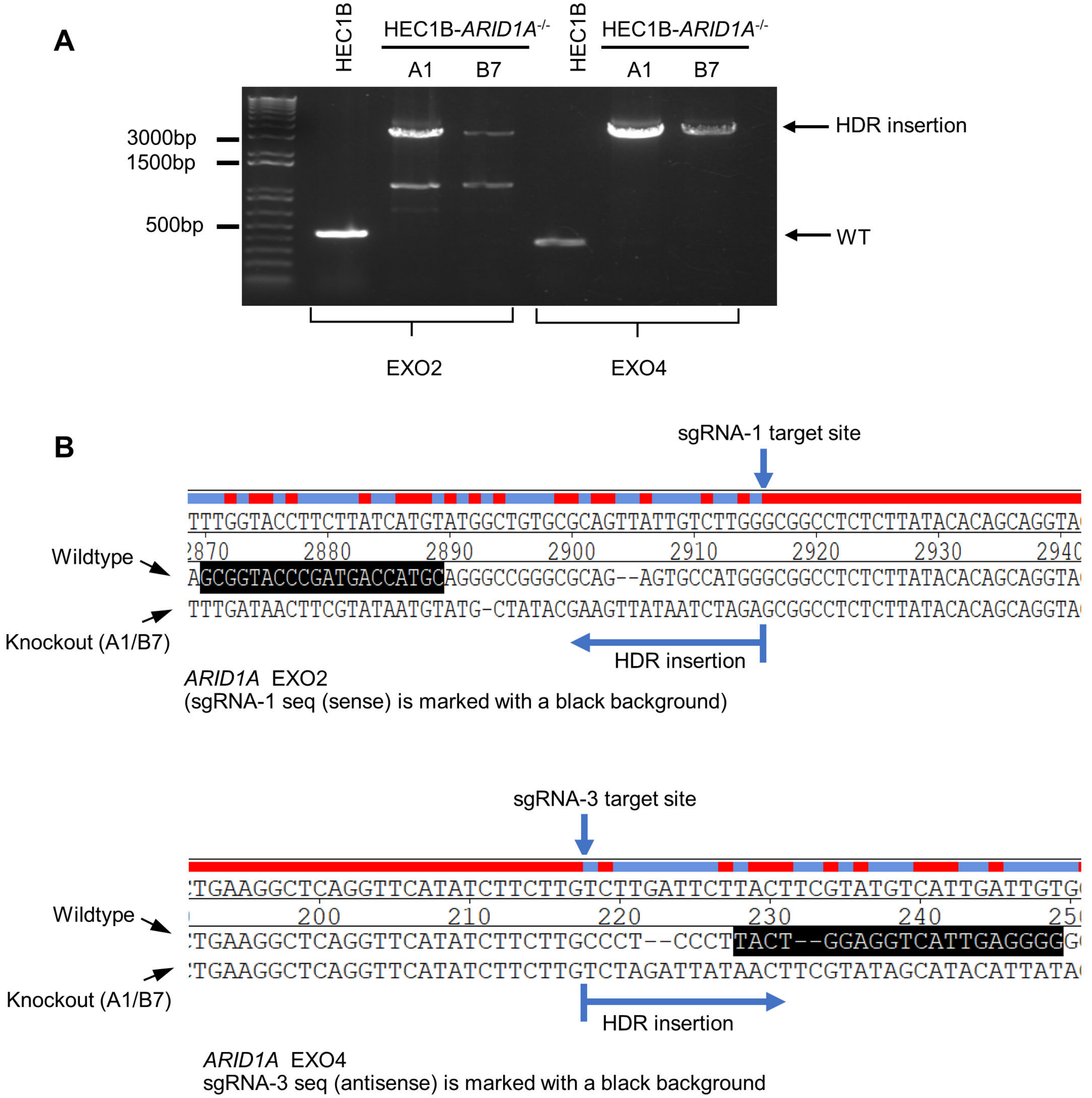

Supplementary Figure S2. Verification of ARID1A-KO in HEC1B cells. A. PCR amplification of *ARID1A* exon2 and exon4 in HEC1B-*ARID1A*$^{+/+}$ and two ARID1A$^{-/-}$ clones. *ARID1A*$^{-/-}$ clone A1 and B7 are *ARID1A* homozygous knockout with a HDR donor plasmid insertion into ARID1A. **B.** Sequencing analysis of the sgRNA target site on *ARID1A* exon2 and exon4 in HEC1B-*ARID1A*$^{+/+}$ and two *ARID1A*$^{-/-}$ clones. SgRNA-1 and sgRNA-2 target site locate in exon2. SgRNA-3 target site locate in exon4. *ARID1A*$^{-/-}$ A1 and B7 both have HDR insertion at sgRNA-1 and sgRNA-3 target site. No mutation was identified in sgRNA-2 target site.

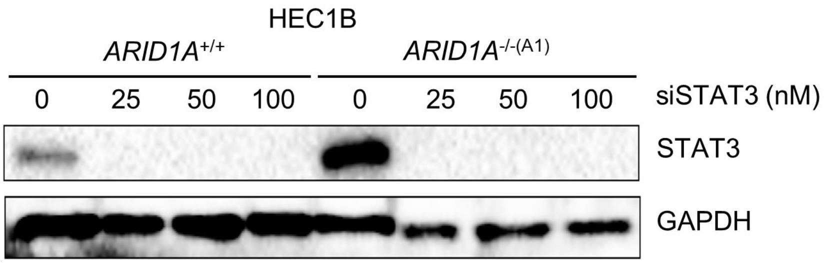

Supplementary Figure S3. Immunoblot analysis of STAT3 expression in ARID1A-isogenic HEC1B cell pair upon siRNA knockdown.

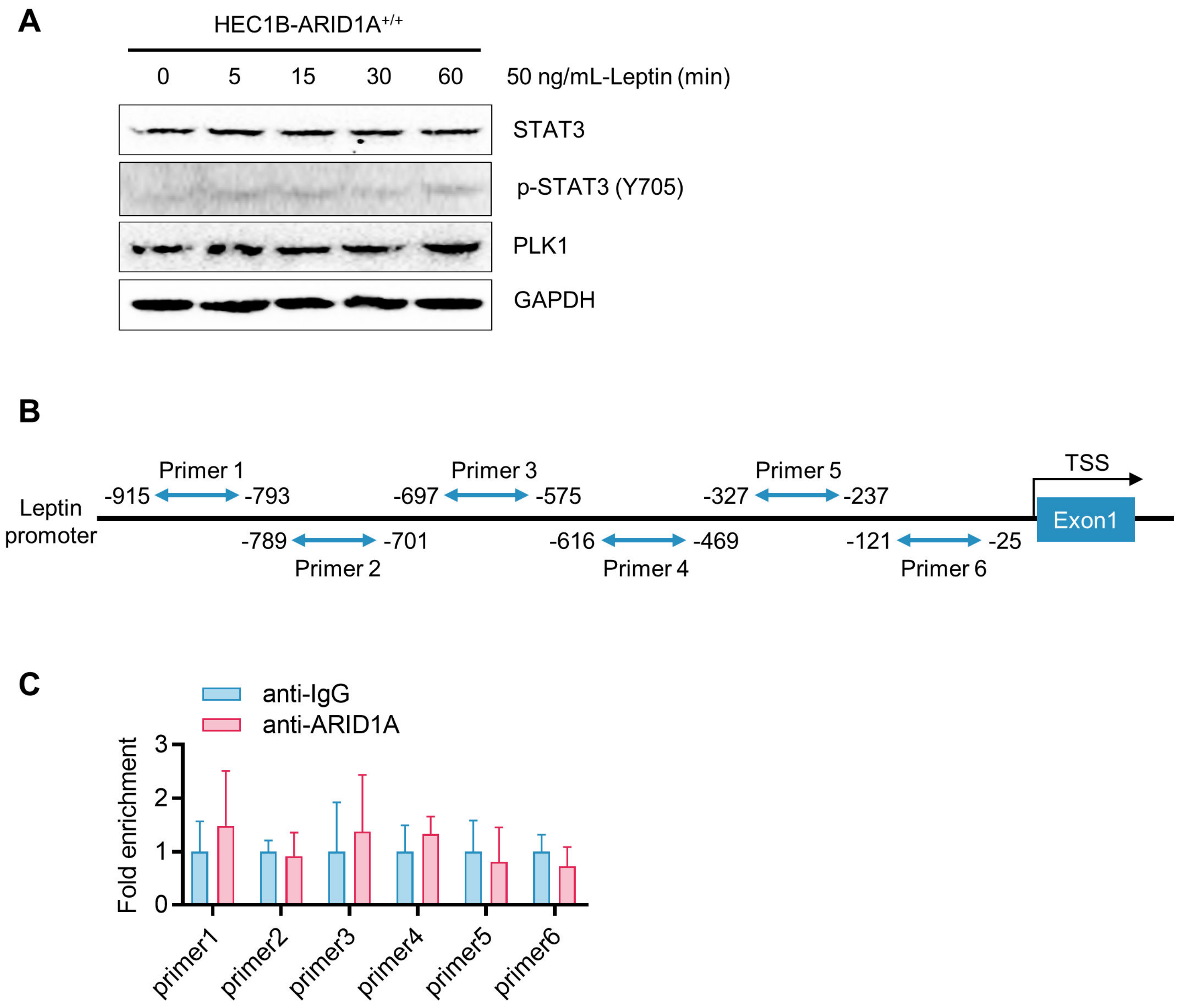

Supplementary Figure S4. Leptin does not activate STAT3 signaling in endometrial cancer cells. A. The immunoblot analysis of STAT3 signaling in the presence of leptin active protein. **B.** The designed primers in leptin promoter area for Chromatin immunoprecipitation (ChIP) experiment. **C.** The ChIP assay was performed using anti-ARID1A antibody. The chromatin was prepared from HEC1B-*ARID1A*$^{+/+}$ cells. IgG was used as a control. Data are mean±SD.

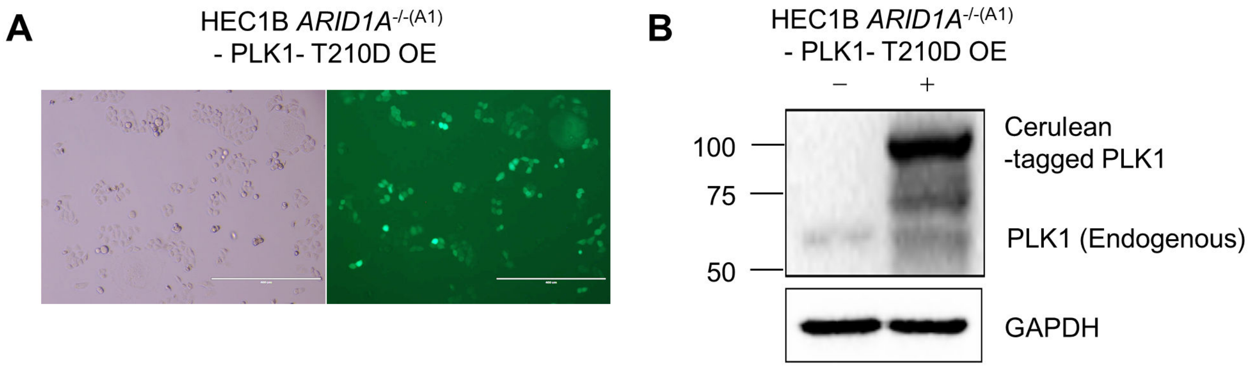

Supplementary Figure S5. Overexpression of a constitutive active form of PLK1 (PLK1-T210D). **A.** The images of the Cerulean fluorescence protein was used to assess transfection efficiency of PLK1-T210D plasmid tagged with Cerulean. Scale bars, 400µm. **B.** The immunoblot analysis of PLK1 after transfected PLK1-overexpression plasmid with a tagged protein Cerulean in ARID1A$^{-/-}$ cells.

Table S1. IC_{50} and selectivity index (SI) values of the top five synthetic lethal drug candidates.

Drug name (target)	IC_{50} (μM, *ARID1A*$^{+/+}$; *ARID1A*$^{-/-}$)	Selectivity index (SI)
HOpic (PTEN inhibitor)	16.70; 2.12	7.88
SKPin C1 (SKP2 inhibitor)	68.46; 17.67	3.87
Stattic (STAT3 inhibitor)	14.50; 5.67	2.56
Gandotinib (JAK1/2/3 inhibitor)	27.48; 11.87	2.32
MK-8745 (AURKA inhibitor)	13.26; 6.52	2.03

Table S2. Sequence information of three sgRNA in the CRISPR/Cas9 plasmid.

SgRNA	Forward (5'to3')
SgRNA-1	GCGGTACCCGATGACCATGC
SgRNA-2	ATGGTCATCGGGTACCGCTG
SgRNA-3	CCCCTCAATGACCTCCAGTA

Table S3. Sequence information of primers designed to amplify the ARID1A exons 2 and 4 containing the 3 sgRNA target sites.

Primer	Forward (5'to3')	Reverse (5'to3')
Exon-2	TGGATCAGATGGGCAAGATG	GCCAGTCAGGTCAAGAGAAA
Exon-4	GAGACAGTCCCATAACCCTTTC	AGGGAGACAGAACAGACATCTA

Table S4. Sequence information of siRNA used in this study.

siRNA	Forward (5'to3')	Reverse (5'to3')
STAT3	rArGrGrGrCrArArArGrGrCrUrUrArCrUrGrAr UrArArArCTT	rArArGrUrUrUrArUrCrArGrUrArArGrCrCrUrUr UrGrCrCrCrUrGrC
OSM	AUAUUAACAUUAAAUAUACAU	GUAUAUUUAAUGUUAAUAUUG
PLK1	rArArUrArUrUrUrCrUrArUrUrGrArArUrUrC	rCrArGrUrUrCrCrGrArArUrUrCrArArUrArG

Table S5. Sequence information of primer used in RT-qPCR.

Primer	Forward (5'to3')	Reverse (5'to3')
GAPDH	GTGGACCTGACCTGCCGTCT	GGAGGAGTGGGTGTCGCTGT
STAT3	ACCAGCAGTATAGCCGCTTC	GCCACAATCCGGGCAATCT
IL6	ACTCACCTCTTCAGAACGAATTG	CCATCTTTGGAAGGTTCAGGTTG
OSM	CACAGACTGGCCGACTTAGAG	AGTCCTCGATGTTCAGCCCA
IL31	GTGCTCGTGTCCCAGAATTAC	TGTCTTGAGATATGCCCGGAT
LIF	CCAACGTGACGGACTTCCC	TACACGACTATGCGGTACAGC
CNTF	GAAGATTCGTTCAGACCTGACTG	AAGGTTCTCTTGGAGTCGCTC
CT-1	AGACCCCCAGACTGATTCCTC	AGCTGCACATATTCCTGGAGC
CLCF1	TTTCAACGAGCCAGACTTCAAC	GAGGCCACGCAAGTAACACA
IL19	TCCTGGCGTTCTACGTGGA	TGACATTGCCGCAGAGTTTTC
IL20	ATGAAAGCCTCTAGTCTTGCCT	GCCCCGTATCTCAGAAAATCC
IL24	TTGCCTGGGTTTTACCCTGC	AAGGCTTCCCACAGTTTCTGG
IL21	TAGAGACAAACTGTGAGTGGTCA	GGGCATGTTAGTCTGTGTTTCTG
CSF3	GCTGCTTGAGCCAACTCCATA	GAACGCGGTACGACACCTC
leptin	TGCCTTCCAGAAACGTGATCC	CTCTGTGGAGTAGCCTGAAGC
IFNα1	GCCTCGCCCTTTGCTTTACT	CTGTGGGTCTCAGGGAGATCA
IFNβ1	ATGACCAACAAGTGTCTCCTCC	GGAATCCAAGCAAGTTGTAGCTC
PLK1	AAAGAGATCCCGGAGGTCCTA	GGCTGCGGTGAATGGATATTTC

Table S6. Sequence information of primer used to analyze ChIP DNA.

Primer	Range from the TSS (bp)	Forward (5'to3')	Reverse (5'to3')
STAT3-Primer 1	−807 to −682	CCAAGTAGGCAAGGTGGAAA	GTACAGGCGAATGCAGAAGA
STAT3-Primer 2	−650 to −515	CTTGAAGTGATGGAACGGAGTA	CCTGCTTTGAACTTCAGTTTCTG
STAT3-Primer 3	−515 to −400	CGGAGTCACCCATGTTCTTT	CTCTTACCACGCGGGAATC
STAT3-Primer 4	−397 to −259	CTCAACCTCGCCACCAC	CCGGAATGTCCTGCTGAAA
STAT3-Primer 5	−262 to −155	CGGTCATCTTCCCTCCCT	CCCTGTGCTGGCTGTTC
STAT3-Primer 6	−136 to −27	GCTCCGCCCTTTCTCCTA	CATGAAAGGCCAGCTCGTT
OSM-Primer 1	−758 to −666	AGGCCCAGAGAGGTTAAGT	GGACACTTGGAGCAAGAGAAA
OSM-Primer 2	−606 to −462	CAGAGGGTGCACGATGG	GGACATCTGTGAGCGGAAA
OSM-Primer 3	−544 to −407	GGGCCTGAACCACAATCT	GAGCGAGACATCTTCCTTCTT
OSM-Primer 4	−397 to −278	CCAACCACCGTGTCCTG	CATGCCATCTTCAGGGAACT
OSM-Primer 5	−290 to −187	GAAGATGGCATGGACCCTTT	CGAATTCGTTCTTCGAGGTCA
OSM-Primer 6	−113 to −13	GGGTCATGTTCCCAGAAGG	GCTGGCAGCCACTTTATG
leptin-Primer 1	−915 to −793	GCTGGACCTTAGATTCCTCATC	GGTAGGCATGTTTCACCTGTA
leptin-Primer 2	−789 to −701	CACTCACAGGCTATGATGACAA	CCTCAACTCCGTATTTCCTACG
leptin-Primer 3	−697 to −575	AGACCCACAGTATGTCCAGA	AATCGAGTTCCAAGCCTCAG
leptin-Primer 4	−616 to −469	GCCAGCCAGAGACAACTT	AGGGAAGCCCAGGGTTA
leptin-Primer 5	−327 to −237	CTCGAAGCACCTTCCCAAG	GCTGGTAGGAGCGAGAAATC
leptin-Primer 6	−121 to −25	GCACGTCGCTACCCTGA	TATAGCGGCCCGATCACAA
